# ‘Fit-for-purpose?’ – challenges and opportunities for applications of blockchain technology in the future of healthcare

**DOI:** 10.1186/s12916-019-1296-7

**Published:** 2019-03-27

**Authors:** Tim K. Mackey, Tsung-Ting Kuo, Basker Gummadi, Kevin A. Clauson, George Church, Dennis Grishin, Kamal Obbad, Robert Barkovich, Maria Palombini

**Affiliations:** 1 Department of Anesthesiology and Division of Infectious Disease and Global Public Health, University of California, San Diego School of Medicine, San Diego, CA USA; 2 Department of Healthcare Policy, Technology and Research, University of California, San Diego – Extension, San Diego, CA USA; 3 Global Health Policy Institute, San Diego, CA USA; 4 BlockLAB, San Diego Supercomputer Center, La Jolla, CA USA; 5 UCSD Health Department of Biomedical Informatics, University of California San Diego, La Jolla, CA USA; 6 Bayer Corporation, 100 Bayer Boulevard, Whippany, NJ 07981 USA; 7 Department of Pharmacy Practice, Lipscomb University College of Pharmacy & Health Sciences, Nashville, TN USA; 8 Department of Genetics, Harvard Medical School, Boston, MA USA; 9 Nebula Genomics, Inc., San Francisco, CA USA; 10 Productive Consulting, Mountain View, CA USA; 11 Health Linkages Inc., Mountain View, CA USA; 12 IEEE Standards Association, 445 Hoes Lane, Piscataway, NJ 08854 USA

**Keywords:** Blockchain, Distributed ledger technology, Healthcare technology, Health informatics, Supply chain, Clinical trials, Medical licensure, Genomics, Electronic health records

## Abstract

Blockchain is a shared distributed digital ledger technology that can better facilitate data management, provenance and security, and has the potential to transform healthcare. Importantly, blockchain represents a data architecture, whose application goes far beyond Bitcoin – the cryptocurrency that relies on blockchain and has popularized the technology. In the health sector, blockchain is being aggressively explored by various stakeholders to optimize business processes, lower costs, improve patient outcomes, enhance compliance, and enable better use of healthcare-related data. However, critical in assessing whether blockchain can fulfill the hype of a technology characterized as ‘revolutionary’ and ‘disruptive’, is the need to ensure that blockchain design elements consider actual healthcare needs from the diverse perspectives of consumers, patients, providers, and regulators. In addition, answering the real needs of healthcare stakeholders, blockchain approaches must also be responsive to the unique challenges faced in healthcare compared to other sectors of the economy. In this sense, ensuring that a health blockchain is ‘fit-for-purpose’ is pivotal. This concept forms the basis for this article, where we share views from a multidisciplinary group of practitioners at the forefront of blockchain conceptualization, development, and deployment.

## Background

### Tim Mackey (Fig. 1)

Whether you are a clinician, researcher, entrepreneur, administrator, or executive, you are probably familiar with the term ‘blockchain’. At its core, blockchain is a new type of digital architecture, consisting of a shared, immutable ledger that can better ensure the resilience, provenance, traceability, and management of health data. It has been hailed as a revolutionary technology, but whether it meets this potential remains to be tested.

**Fig. 1:**
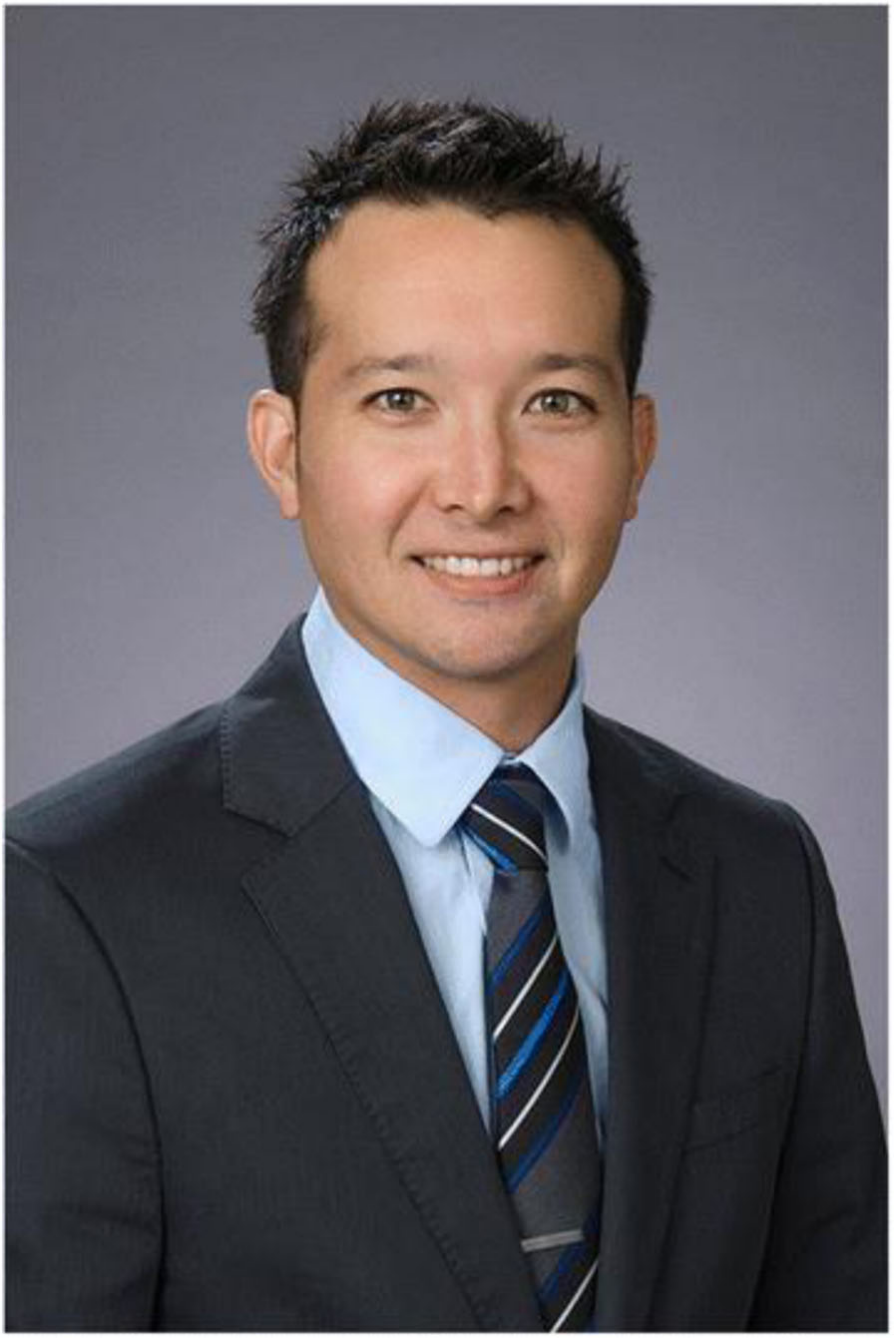
Tim K. Mackey is an Associate Professor at UC San Diego School of Medicine, the Director of Healthcare Research and Policy at UC San Diego Extension, and the Director of the Global Health Policy Institute (www.ghpolicy.org). He has a multidisciplinary background, with a research focus on global health policy, law, governance, innovation and technology, and has worked or consulted for organizations including the World Health Organization, the US Department of State, and the US Department of Justice, among others. He is also the co-Chair of the IEEE Standards Association Supply Chain/Clinical Trials Technology Implementation Industry Connections Program, which focuses on stakeholder collaboration around blockchain technology for the pharmaceutical supply chain and is also a Lab Principal Faculty with the BlockLab at the San Diego Supercomputer Center

To understand blockchain’s potential for healthcare, we first need to understand some basic technical elements. Unlike traditional centralized databases, data on a blockchain can be distributed across multiple databases/computers (also known as ‘nodes’) so that everyone has the same copy (ledger) of a transaction [1]. ‘Blocks’ of data are linked together by a hash (a digital signature of random letters and numbers) to form a ‘chain’ of data that contains the complete history of the transaction and renders it tamper resistant [2, 3]. Blockchain data is also secured through cryptography (advanced encryption) so that participants can trust that ‘blocks’ of data are authenticated and verifiable [2]. These technological features result in decentralized data systems (not held by a central authority vulnerable to breach or potentially acting as a single point of failure), represent a single source of information among all participants, and have inherently higher levels of trust (as transactions are immutable, secure, and subject to consensus of the participants). Blockchains can also be permissioned to limit participation and access to or sharing of data [4]. Finally, there are public blockchains (e.g., public network-based blockchains such as the popular cryptocurrency Bitcoin) and private or ‘business’ blockchains (e.g., private networks not open to the public but instead used by a select group of trusted participants) [5]. Some popular platforms for blockchain deployment include Ethereum and Hyperledger, though offerings are growing.

**Fig. 2:**
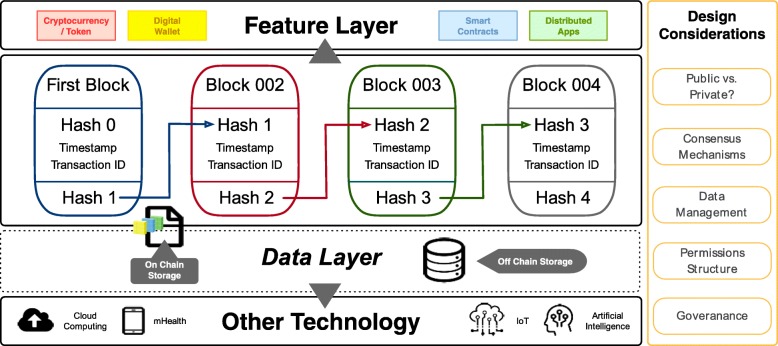
Depiction of blockchain data architecture components. This includes the core functions of blockchain data by generation of a first (genesis) block that is timestamped and may include certain transaction data/metadata (transation data) or state-of-data information. These blocks of data are chained together via a cryptographic hash of the data. The data layer represents where data can reside on the blockchain, primarily either storing data on the blockchain itself (on-chain storage) or storing the data in a different source but including a pointer or using a distributed application as an intermediary (off-chain storage). The core functions of the blockchain should also assess certain design considerations (in far right yellow box), including whether the blockchain is public, private or consortium, the consensus mechanism to be used, the type of permissions structure, where data should reside and how it should be managed, and the governance of the blockchain (who are the users, peers, validators, nodes, etc.). Finally, a feature layer including blockchain-enabled technology options, such as the use of cryptocurrencies/tokens, digital wallets, smart contracts, and distributed applications, can also be added if needed for a particular healthcare use case

Beyond these core features, blockchains can also enable other technologies such as distributed applications (those that run on multiple computers in a network) and smart contracts (computer code that can execute terms of a contract between parties) as well as the use of cryptocurrencies (digital or virtual currencies) [2, 6–8]. Blockchains can also act as a digital backbone for other technologies able to interface with blockchain systems such as cloud computing, artificial intelligence, eHealth and mHealth devices/applications, and the broader Internet of Medical Things (IoMT) [6, 9, 10]. Thus, the blockchain environment is expansive and modular, and has the flexibility to be adopted for various use cases in healthcare and beyond (see technology architecture summary in Fig. 2).

**Fig. 3:**
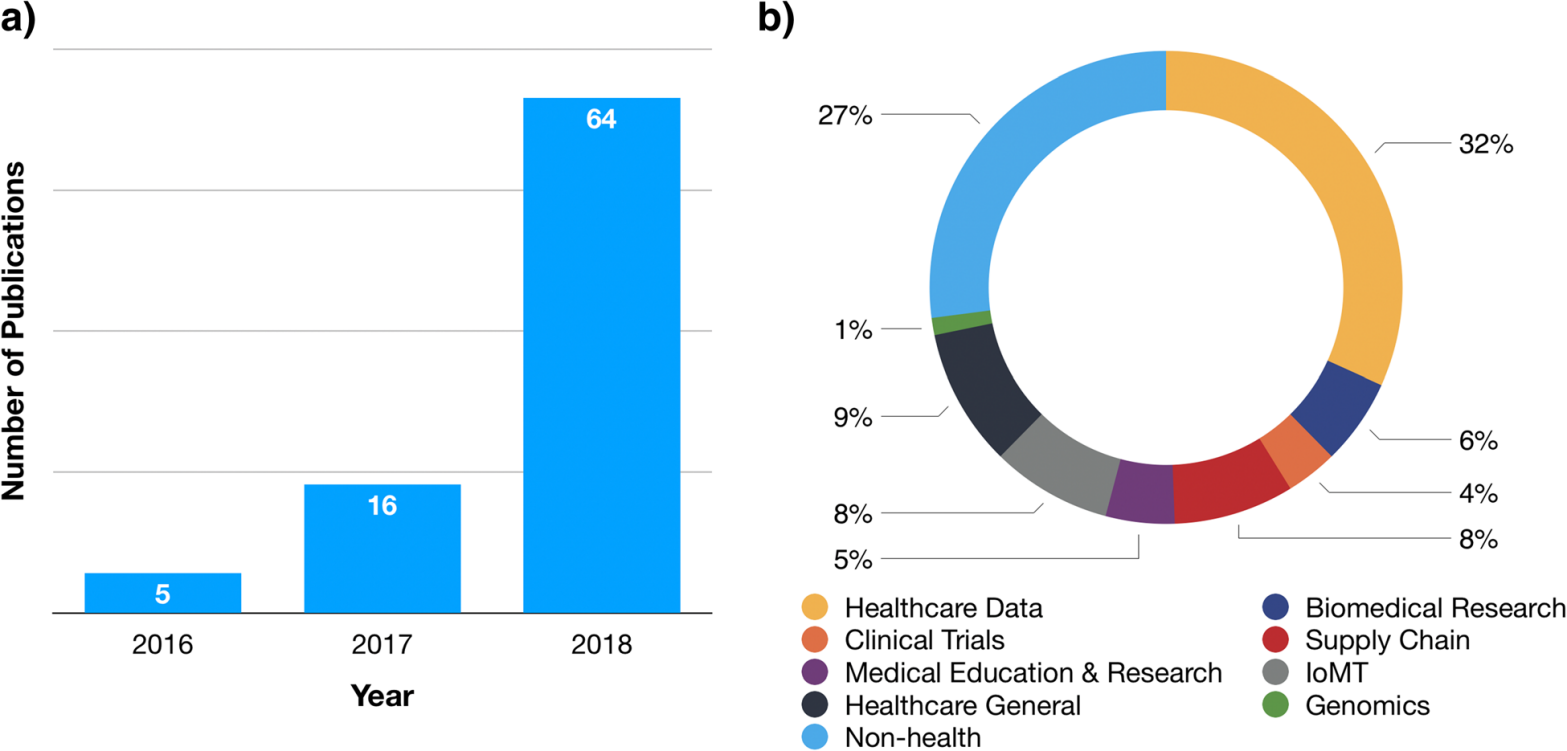
Summary statistics of publications with the term ‘blockchain’ in the Title or Abstract field in PubMed Central (as of December 1, 2018). **a** A depiction of the increase in the number of blockchain publications from 2016 to 2018. **b** A breakdown of the health and life science categories of blockchain publications, including healthcare data, clinical trials, medical education and research, supply chain, biomedical research, genomics, Internet of Medical Things (IoMT), and general articles about blockchain applications in healthcare. Non-health articles included those discussing blockchain in other industries such as energy, finance (cryptocurrencies), non-health supply chains, Internet of Things (non-health), news and media, and ecology

Reflecting the increased attention given to blockchain in healthcare and life sciences, the number of PubMed indexed articles including the keyword ‘blockchain’ in the title or abstract fields has increased dramatically, from only 5 in 2016 to 64 in 2018 (Fig. 3). The published papers evidence the wide variety of use cases that are being researched for health blockchains, including management and interoperability of healthcare data (e.g., patient healthcare, consumer health, and hospital data), improving integrity of published research, clinical trial management, use and integration into IoMT applications (including mHealth and remote patient monitoring), advancing genomics and precision medicine, applications for biomedical and medical education and research, pharmaceutical supply chain management and security, implications of blockchain for global health, and general articles of the various opportunities for blockchain in medicine, engineering and the life sciences [3–5, 7, 11–24]. However, it is important to note that the published literature represents only a snapshot of global blockchain activity, as many health-related blockchain projects are published in white papers, news articles, press releases, presented at conferences, or are otherwise undisclosed as they are developed for commercialization purposes. Several large technology firms, such as IBM, Intel, and Microsoft, are heavily invested in blockchain technology development, and the World Economic Forum estimates that, by 2025, 10% of the global gross domestic product will be stored on blockchain technology [25].

Despite blockchain’s potential as an emerging technology to be innovative and disruptive, it remains immature, particularly in healthcare. According to Gartner [26], blockchain technology is in a ‘hype cycle’ characterized by stages of innovation triggers, inflated expectations, disillusionment, enlightenment, and ending in a “*plateau of productivity*”, with healthcare and life sciences currently squarely in the middle of phase one and two of the curve. Reflecting the fact that it is still early days for health blockchains, there are few real-world examples of blockchain systems that have gone into production and that also have strong commercial or user adoption in healthcare. In contrast, other sectors of the economy have seen much faster adoption, including financial technology services and supply chain and logistics.

Despite the core characteristics of decentralization, security, provenance, transparency, trust, and better management of data being clear benefits to address acute healthcare needs, an approach that ensures that blockchain technology is ‘fit-for-purpose’ for specific and diverse healthcare challenges is required. Importantly, when assessing the viability of a blockchain for health, what core blockchain characteristics and design principles need to be taken into account, and how can they address the real-world legal, regulatory, privacy, business, and provider and patient-centric considerations unique to healthcare?

In an attempt to address these challenges, this Forum article presents a ‘fit-for-purpose’ health blockchain design framework that includes fundamental questions regarding basic blockchain design principles, data sharing and management, and decisions about governance, as well as exploring the technologies that can be used to enhance blockchain function and defining the ultimate goal for the blockchain solution. If these questions can be appropriately mapped, then there is a higher likelihood that the blockchain approach will be ‘fit-for-purpose’ for whatever healthcare challenge has been identified. The framework questions are based on six principles, as follows:

1. **Blockchain design types:** Decision of whether your blockchain design will be a public blockchain (generally open to participation by anyone and not permissioned), private blockchain (involving limited participation and having permission structures), or a hybrid (blockchain systems with both public and private designs).
2. **Data sharing and access:** In healthcare, sharing and access to health-related data is subject to various privacy, legal, and regulatory requirements (such as the Health Insurance Portability and Accountability Act (HIPAA) and the General Data Protection Regulation (GDPR)). Decisions need to be made about what type of data will be shared with and among participants, if any, whether data will be stored on-chain, off-chain or on a side-chain, and the type of permission structures that will be utilized.
3. **Decisions about blockchain governance:** Governance is a crucial component to the design of a blockchain system. The nodes, users, peers, and/or validators of the blockchain will need to be defined, as well as whether it will be comprised of only trusted partners, a consortium of participants, or participation of public entities or regulators, and include patients/consumers/the public. Finally, how these actors will make decisions about how to govern the blockchain (including choices regarding consensus mechanisms, permissions, and data governance) will also need defining.
4. **Added technology to enhance blockchain function:** As previously discussed, the blockchain architecture can also enable the use of other technologies, including the development of an application layer that interfaces with the blockchain, the use of smart contracts to automate processes when certain agreed upon conditions are met, and the use of a cryptocurrency/tokens to incentivize participation that ideally provides shared benefits to all participants.
5. **Ultimate healthcare goal of the blockchain:** Although it may seem obvious, a critical issue that must be addressed is the definition of the ultimate goal of the blockchain to improve healthcare. Beyond the core benefits of a distributed, immutable, transparent, and higher trust system, the unique benefits a blockchain system can provide for healthcare processes over other existing technologies must also be assessed. Not all blockchains will have the same goal(s). For example, some may be designed to simply lower healthcare transaction-related costs by improving and automating processes (such as the use of smart contracts), removing intermediaries, or reducing administrative burden. Others may focus on creating mechanisms to drive revenue generation. Some will prioritize enabling better data collection, use, and sharing from patients, consumers, and providers through the offer of incentives (such as tokens). Further, others may focus on more indirect benefits such as increasing compliance or preventing fraud. Eventually, some blockchains may be designed to achieve multiple goals, yet may start with the most pragmatic use first.
6. **The need for a blockchain:** A final question may simply ask whether the healthcare-related challenge or goal really needs a blockchain, or if it can be better facilitated by another form of technology.

Though the above ‘fit-for-purpose’ blockchain framework considerations are not exhaustive, they form a basis for thinking about how blockchains can be designed in ways that have shared goals of improving healthcare and ultimately patient outcomes. Using this framework as a starting point, this article introduces a set of prominent use cases in healthcare to further examine what a ‘health’ blockchain may look like in the near future based on ongoing research, published studies, and real-world examples. The aim of this article is to explore different perspectives about key design elements, challenges, opportunities, and best practices for the future health blockchain landscape. To accomplish this, the article brings together a diverse and multidisciplinary group of experts from academia, the private sector, healthcare startups, and professional technology associations to discuss use cases in healthcare records, clinical trial management, medical credentialing and licensing, genomics and precision medicine, pharmaceutical supply chain, and biomedical research. It closes with a discussion from the IEEE Standards Association about the importance of setting technical and industry standards to ensure blockchain in healthcare moves forward and realizes its potential as a revolutionary force for 21st century healthcare.

## Using privacy-preserving predictive models and blockchain technology for electronic health records

### Tsung-Ting Kuo (Fig. 4)

Healthcare record management is one of the most important application domains for blockchain technology [4, 19, 25, 27]. Blockchain healthcare record management focuses on the sharing of data across different healthcare stakeholders, while preserving the source, provenance and, oftentimes, privacy of such data in a way that can enable more powerful data analysis and insights from population health analytics [25, 28–34]. Among healthcare record applications, this section focuses on an example of blockchain-based privacy-preserving prediction modeling that leverages many of the strengths of blockchain technology [35–37].

**Fig. 4:**
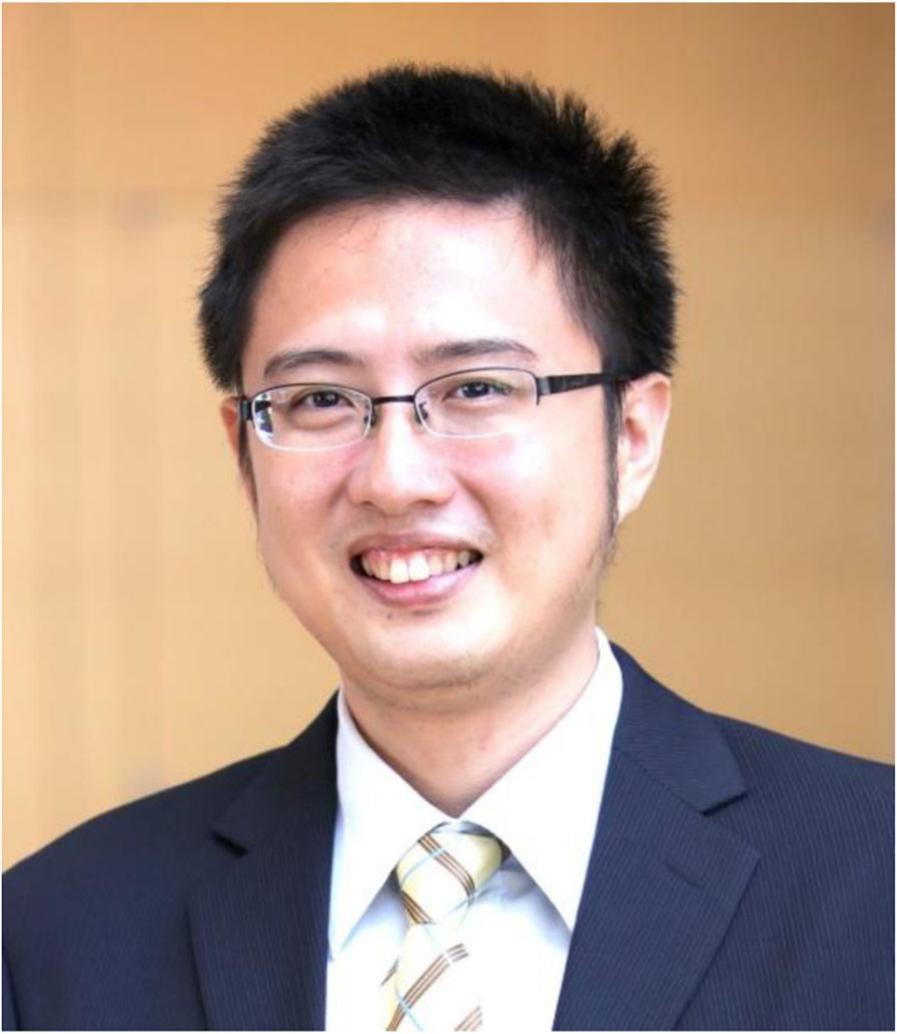
Dr. Tsung-Ting Kuo is an Assistant Professor of Medicine at the University of California San Diego (UCSD) Health Department of Biomedical Informatics (DBMI). He earned his PhD from the National Taiwan University (NTU) Institute of Networking and Multimedia. Prior to becoming a faculty member, he was a Postdoctoral Scholar in UCSD DBMI and received the UCSD Chancellor’s Outstanding Postdoctoral Scholar Award. He was a major contributor towards the UCSD DBMI team winning the Office of the National Coordinator for Health Information Technology healthcare blockchain challenge in 2016, and also the NTU team winning the Association for Computing Machinery Knowledge Discovery and Data Mining Cup competition four times. He was awarded a NIH Pathway to Independence Award (Parent K99/R00) for a blockchain-based biomedical and healthcare study. His research focuses on blockchain technologies, machine learning, and natural language processing

In this specific use case, hospitals or healthcare institutions aim at training a machine learning model from the healthcare records stored in their electronic healthcare record (EHR) systems, and then using the learnt model to predict patient outcomes (e.g., the risk of having a certain condition or disease). Specifically, while targeting rare conditions/diseases (e.g., Kawasaki disease), there may not be enough patient records for a single hospital/institution to learn a generalizable model solely from its own EHR data. To solve this problem, intuitively, hospitals/institutions can share their data to enlarge the number of records; however, sharing patient data directly may lead to privacy risks such as re-identification [38] and data breaches [39]. Therefore, several privacy-preserving prediction modeling methods have been proposed [40, 41], enabling the hospitals/institutions to collaboratively train a predictive model by sharing only partially trained machine learning models (i.e., a set of aggregated parameters) instead of patient-level records.

However, these state-of-the-art methods are mainly centralized (i.e., client-server architecture), which may lead to several concerns such as the single-point-of-failure on the server. To mitigate these issues, the combination of blockchain and privacy-preserving prediction modeling provides a solution for the hospitals/institutions to collaborate and train a generalizable predictive model without exchanging patient-level data, while obtaining the benefits of a distributed (i.e., peer-to-peer) architecture without architectural concerns (e.g., single-point-of-failure). In this blockchain-based solution approach, the users are the hospitals or healthcare institutions participating in the cross-institutional model learning. The data that users’ input is the patient-level data from the EHR, with the same format and semantic meaning. Importantly, direct data is not shared across hospitals/institutions, with only the predictive models learnt from the EHR data being shared via the blockchain network. In such a blockchain network, the peers are actually the same as the users.

To ‘validate’ (train a predictive model collaboratively in this use case) the data (i.e., models), each peer encloses their partial models in the transactions (at the transaction metadata level, as shown in Fig. 2) to create blocks, retrieves the models from other peers, and then updates the model using their own EHR data. The learning process is conducted in an ‘online’ fashion, such that the model is updated using only partial data in a sequential order. Additionally, training errors are used to guide the order of the online learning on the blockchain, based on an intuition that the site containing data with higher error may provide more information to improve the model. This iterative learning process is repeated until a consensus predictive model for all peers is identified. In this way, blockchain provides specific benefits for problem solving such as protecting privacy by exchanging models only, avoiding the single-point-of-failure, and generating immutable logs for the learning process.

Following this Forum’s framework of the ‘fit-for-purpose’ blockchain design elements, the design type of the privacy-preserving learning based on blockchain is private. In terms of data sharing, the models and their meta-information (e.g., the local training error of a model) are shared on-chain, while there is no off-chain data sharing. For governance, only participating hospitals and institutions are included in the blockchain network, and the incentive of these users/peers to participate is the improved predictive power of the models to glean new insights into patient and population health outcomes.

Ultimately, the goals of this blockchain-based learning method for health records management include supporting comparative effectiveness studies, biomedical research, and eventually patient care.

## Blockchain-enabled medical professional credentialing and licensing

### Kevin A. Clauson (Fig. 5)

**Fig. 5:**
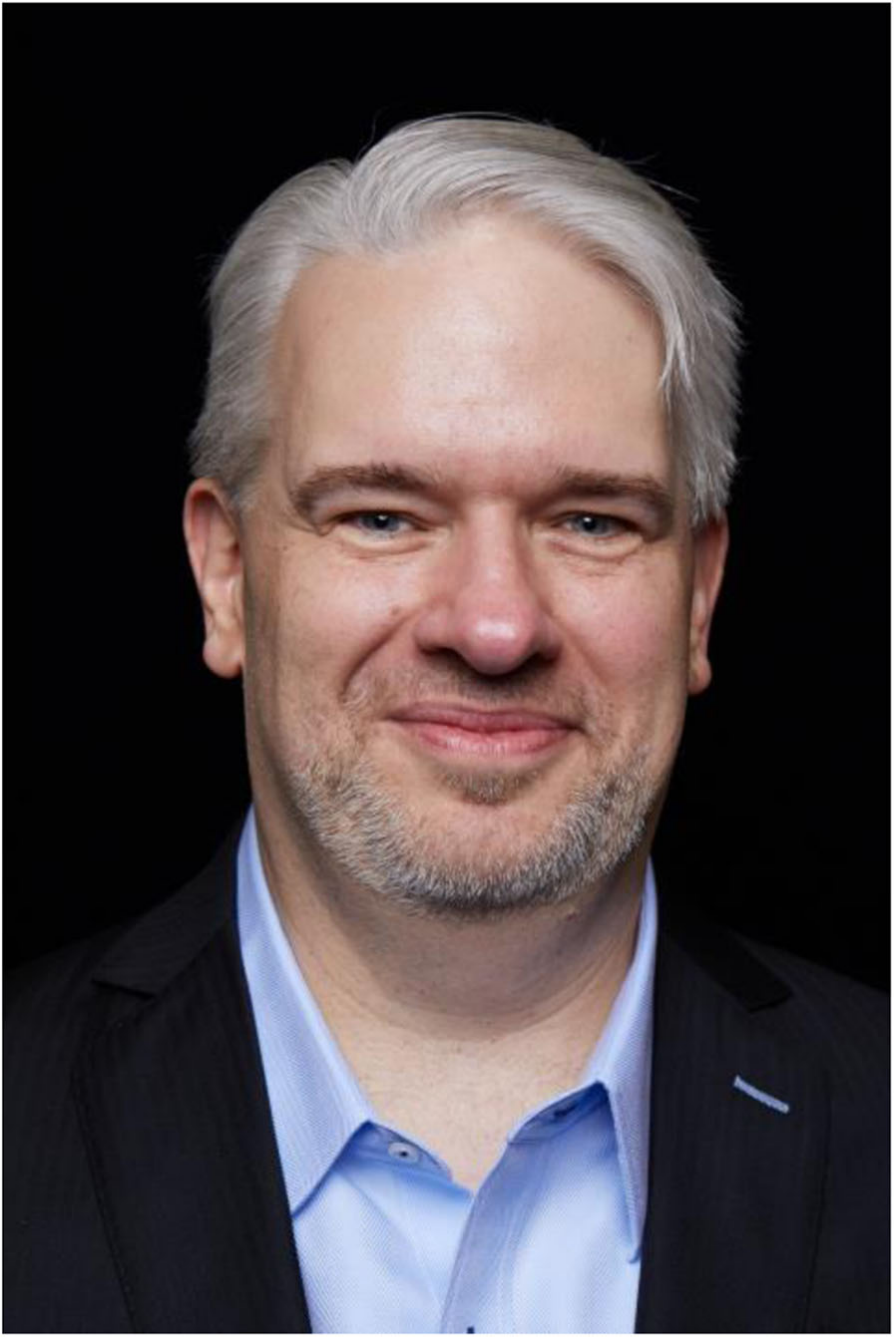
Kevin A. Clauson is Associate Professor of Pharmacy Practice at the Lipscomb University College of Pharmacy & Health Sciences. His investigation of blockchain for the health supply chain began in 2015 as a follow-up to his previous role as director of a World Health Organization Collaborating Center. He has received the Blockchain for Education Collaboration Award, recognizing the partnership between Lipscomb University and Hashed Health to build an Ethereum-based platform to allow degree verification. His research is focused on digital health and his work has generated coverage by the *New York Times*, *Forbes.com*, *The Wall Street Journal*, and *BBC Radio*

Processes for the management of healthcare workforce competencies, including degree verification, credentialing, and continuing education, are largely based on legacy systems that can suffer from inefficiencies, lack of transparency, and avoidable costs [42, 43]. These challenges are further exacerbated by bad actors, as highlighted by a survey of hiring managers (*n* = 2532) that found 28% of observed job candidates claiming unearned academic credentials [44]; this occurs across sectors, including high profile cases like a nationally known US college football coach resigning days after his hire after it was unearthed that he had falsified a master’s degree in education [45]. In a more troubling case, a man falsified both degrees and transcripts to secure a federal leadership position for a technology role in law enforcement and security [46]. Another recent paper documented that 89% of human resource and risk experts reported candidates misrepresent information on applications [47]. Thus, the dual threats of suboptimal processes and bad actors have negatively transformed the credentialing arena from a seemingly mundane activity to a time-intensive, cost-inflated pursuit that can even negatively impact patient safety [48].

Professional credentialing and licensure is also a critical function of most highly regulated sectors of the economy, including multiple actors in the healthcare system. Since the early 1960s, hospitals have been required to verify the competency of physicians in their institutions [49]. In general, medical credentialing of hospital staff and physicians can be time-consuming and, if processed incorrectly, can lead to increases in costs (including disrupting a hospital’s revenue cycle), potential legal liability, and inhibit the ability of a health system to process reimbursements [50, 51]. Included in the process is independent verification of certifications, licenses, qualifications, education, relevant training (e.g., continuing medical education), board certifications (if applicable), relevant accreditations, practitioner’s employment history and, in the case of the US, querying the National Practitioner Data Bank for negative information. Further, providers may maintain independent credentials with multiple systems based on their admitting privileges and may also have practiced in another state [52].

In its simplest terms, a blockchain medical credentialing solution would act as a decentralized data directory where data on the provider’s identity could be verified to its trusted source and continuously updated and reconciled to ensure trust and confidence in the provider’s ability to practice. One model envisions accreditors, hospitals, medical schools and other educational institutions, licensing boards, national health agencies, and other sources of credentialing information serving as nodes and participants in the blockchain (potentially reducing the need for medical credentialing services and other intermediaries). Other models would position a blockchain structure that served as either interstitial or as second layer solutions that could provide connectivity for all of the current data silos involved in credentialing. Analogous to the ‘fit-for-purpose’ model that leverages blockchain enhancement for health supply chain management (as discussed later by TKM and MP), medical credentialing has similarly distributed sets of stakeholders that require varying levels of permissions across the assets’ lifecycle (e.g., in this case, the information relating to a provider’s identity).

One such credentialing lifecycle tool that has been conceived is the Comprehensive Learner Record, which includes coursework, degree(s), competencies, co-curricular activities, experiential learning, microcredentials, and professional (e.g., medical) credentials that could map into licensure and continuing medical education [53]. The Comprehensive Learner Record also includes the Open Badges functionality, which in turn is data aligned with Blockcerts standards. Blockcerts has separately been advanced as an “*open standard for blockchain credentials*” [54]; notably, initial Blockcerts support was for public (e.g., Bitcoin and Ethereum) blockchains, with expansion to other blockchains outlined in their roadmap.

Reflecting again on the ‘fit-for-purpose’ model, this use case can be characterized as individual centric rather than process centric. As such, identity is central to all of these processes, but can be approached via public, private, hybrid, or consortium designs. One such example of a public design is through the Decentralized Identity Foundation, which aims to use an Identity Hub architecture [55] to help accomplish this goal.

Within this broader framework, efforts to leverage the strengths of blockchain to secure provider identities and credentialing include private (e.g., Piper Jaffray), government (e.g., State of Illinois), commercial (e.g., Professional Credentials Exchange), and educational (e.g., Lipscomb University) entities [2]. One collaborative pilot between state government and industry in this area proposes a blockchain-based registry for data sharing, but also adds smart contract functionality to ‘automate workflow’ for medical licensure tied to more than one state in order to further enhance efficiency and reduce costs [56].

While educational credentialing remains limited in scope and onerous to conduct for many institutions of higher education, and medical credentialing and licensure can be disproportionately resource intensive, thoughtfully designed blockchain-based systems and the enhanced functionality of smart contracts offer promise as a means to contemporize these fundamental but antiquated elements of education and healthcare.

## Can we use blockchain to improve clinical trial management?

### Basker Gummadi (Fig. 6)

“*If you have a hammer, everything looks like a nail*” – technology professionals tend to assume their favorite technologies are the solutions for virtually any problem. The ‘hammer’ bias in technology has always been there, and the hammer keeps changing based on the latest trend, with currently trending technologies being blockchain, artificial intelligence, and robotic process automation [57]. However, a better approach is to identify the business problems that need solving and subsequently to evaluate the available technologies, tools, and solutions, while also assessing the attributes and features that are a ‘perfect fit’ to solve that problem. No single tool or process is a ‘silver bullet’ or ‘golden hammer’; it is the combination of the different technologies and tools, selected based on the problem being tackled, and their individual attributes, that are best suited for problem solving. This approach ties in well to the ‘fit-for-purpose’ framework that views blockchain technology as modular and with different technology features that can be customized; it also applies to blockchain development in healthcare verticals like clinical trial management.

**Fig. 6:**
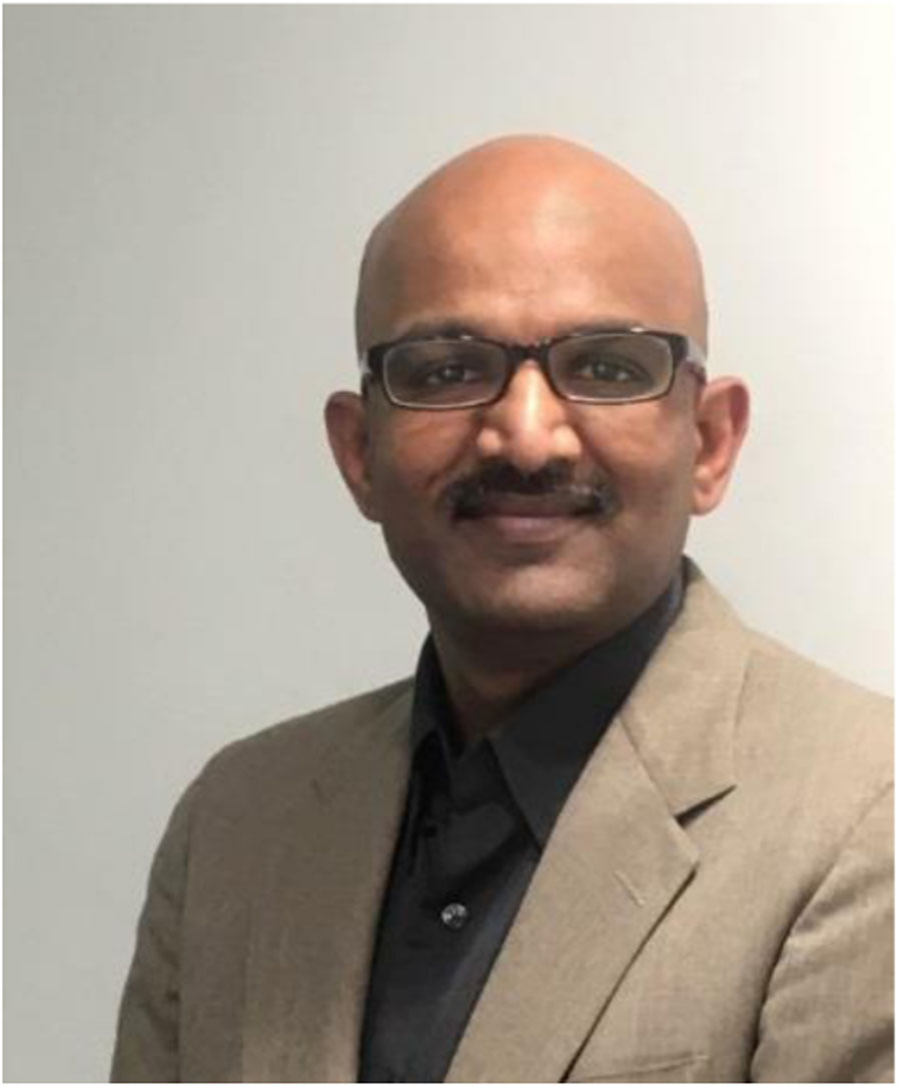
Basker Gummadi is the IEEE Lead for Digitalization of Clinical Trials. He works at Bayer Corporation as Digital Innovation Senior Manager/Deputy Director, and leads a community focused on bringing together stakeholders in the pharma industry and academia to identify business cases for clinical trials using blockchain, artificial intelligence, robotic process automation, and big data. He is the current team lead for projects focused on designing clinical trials to be more patient centric using blockchain technology. Prior to joining Bayer, Basker held various positions in the pharmaceutical industry, including Business Solution Manager at Hoffman-La-Roche, Assistant Director at Schering Plough, and Senior Analyst at Bristol Myers Squibb

The benefits for blockchain in clinical trials management includes moving stakeholders into a distributed network with processes that can be more efficient when you eliminate the need for intermediaries [16]. This domain of healthcare is ripe to leverage core benefits of blockchain technology such as transparency, disintermediation, immutability, auditability, and trust. From a business perspective, a typical clinical trial process is expensive and involves numerous stakeholders [58]. Thus, blockchain technology can help clinical trial sponsors, patients/subjects, principal investigators and site administrators that conduct the clinical trial, Institutional Review Boards, and regulatory authorities.

Once we have identified the different actors involved in clinical trial management, we can then identify challenges and map blockchain solutions based on a ‘fit-for-purpose’ approach. Some of the most pressing challenges in clinical trials include (1) access and management of clinical trial data; (2) data integrity and provenance for clinical trial processes for regulatory purposes; (3) updating and maintaining patient consent; and (4) patient recruitment. Below, some blockchain approaches to address clinical trial management challenges are described, which also illustrate the variety of blockchain designs.

Arguably, the most important stakeholder in a clinical trial is the patient; currently, when a patient leaves a clinical trial, they rarely have access to any of their clinical trial results. If a sponsor wants to share their clinical trial data with a patient, they can easily do so without a blockchain, but it is more beneficial for the patient to be part of a network where multiple sponsor companies share data on the network and data is verifiable to the patient’s identity [59]. On a blockchain platform, additional sources of data, such as from hospitals, care providers, genomic data, proteomic data, and other medical data (e.g., from medical devices), can be added by enabling the respective data providers or systems to share the data with the patient in a blockchain-enabled digital wallet or other patient-centric database.

More robust data access can also enable better patient recruitment into clinical trials. With recruitment costs ranging between US$ 2 billion and US$ 3 billion (also depending on the phase of the clinical trial), this represents a major barrier that continues to increase [60]. Blockchains can aggregate patient and trial data that is anonymized or else subject to patient-driven permissions [59]. In this way, patients and sponsors/sites could connect better with eligible patient populations where there is mutual interest for trial participation. With this challenge, permissioned-based blockchains with the patient at the center of data governance might be the best approach.

Data integrity and data provenance are key in clinical trials. Sponsors and investigational sites have to prove data provenance and respond to queries from regulatory authorities to help ensure that clinical results maintain their integrity from data capture through to interim and final analyses. This process is burdensome and time consuming and increases the costs of a clinical trial’s data sharing and management procedures [61]. Blockchain has an architecture that can transparently show the provenance of the data from the origin to the final clinical summary report. The underlying trust in the data is enhanced, accelerating the regulatory approval process, and regulatory authorities will be better equipped to evaluate clinical trial results and determine if a treatment is safe and beneficial to patients. With regards to clinical trial data management, the design of a blockchain will likely be on a private network, with only trusted nodes associated with the study protocol. In the event of a regulatory inquiry, private key management could also enable a regulator to inspect the data for integrity [61].

Another challenge arises when sponsors are planning a clinical study, as the protocol often goes through several revisions and is revised even after patient enrollment to provide the best outcome for the patients. Sites managing the trial have to ensure appropriate patient consent (often via paper format) with the latest version of the protocol, which is a challenge as consent collection is a dynamic process. Sponsors are accountable for this process, and it is a key area of focus for regulatory authorities in their inspections. In response, sponsors can build a consent workflow using blockchain to implement a process allowing for collection of patient informed consent (including, potentially, e-consent through smart contracts), which is bound to protocol revisions. This process would allow for a built-in layer of transparency and traceability by time-stamping each step of a patient’s consent and potentially automating it via set rules in smart contracts.

The fundamental design elements of clinical trial blockchains requires a private blockchain. In this model, the different stakeholders of sponsors, sites, regulatory authorities, and data providers (claims, proteomic, genomic, hospitals, and physician’s offices) can all be nodes, and patients can act as the participants. In terms of data sharing, the clinical trial data will likely reside within the respective company’s database; the patient will be able to access their data via a pointer to their data on-chain through a unique hash, enabling the patient to have control of their data and share their clinical trial results.

For shared governance of a clinical trial blockchain, only screened and selected patients who are permissioned, with similarly permissioned institutions, would be included in the blockchain network. The incentive for these users/peers is the improved predictive power of the models associated with clinical trial data, collaboratively trained in a distributed and privacy-preserving way (as discussed by T-TK above).

Ultimately, clinical trial blockchains show immense promise to be one of the first successful use cases entering production and show real-world benefits in the healthcare vertical. Shared value, lowering costs, enhancing regulatory compliance, and streamlining clinical trial processes all position blockchain as a critical tool in the future of clinical trial management.

## Blockchain technology to advance biomedical research?

### Robert Barkovich (Fig. 7)

**Fig. 7:**
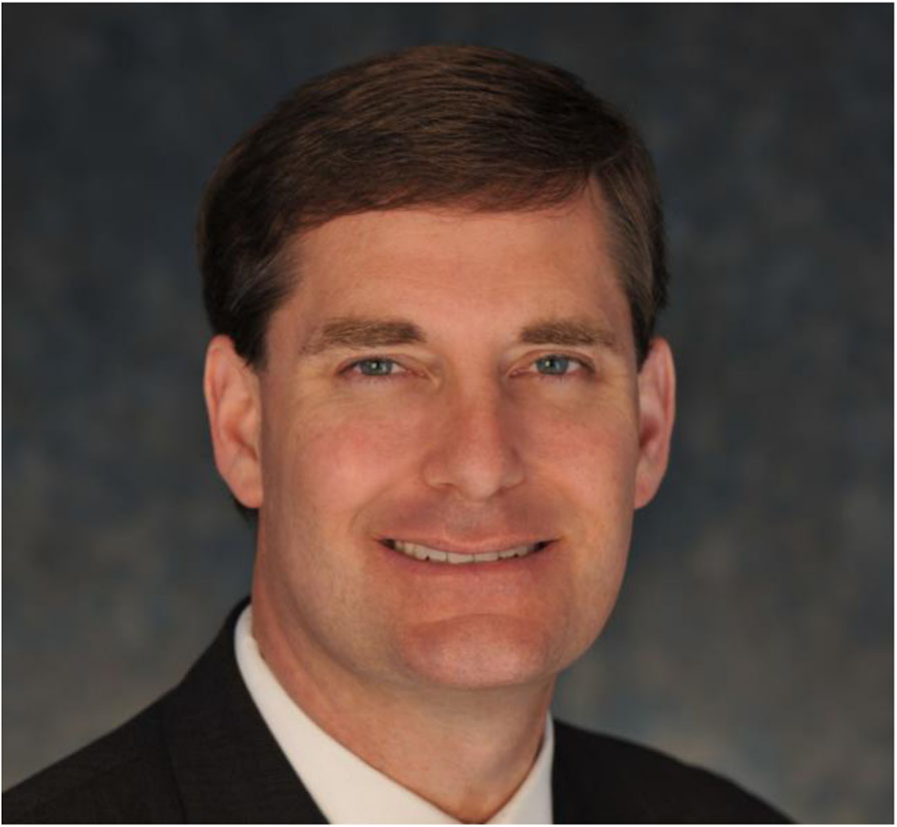
Robert Barkovich is a product leader and entrepreneur with more than 15 years of experience on the software and IT side of healthcare and life sciences. Robert runs Productive Consulting and is the founder of Health Linkages Inc., a company focused on solving the data provenance problem in the healthcare and life sciences industries

Blockchain is being heralded as a breakthrough technology for healthcare and the life sciences. As an example, the Icahn School of Medicine at Mount Sinai in New York opened a Center for Biomedical Blockchain Research in July of 2018 [62]. Nevertheless, there remains much skepticism about blockchain in these industries, in particular regarding how it can solve real-world problems and whether there will be more broad-scale adoption. Blockchain technology shows great promise for biomedical research as it has the potential to address many long-standing challenges in the field. These issues include authenticity and integrity of data, data provenance, consent, data privacy, and data sharing. It also has the potential to enhance efforts towards promoting ‘open science’ to enable transparency, accessibility, reproducability, and mobilization of scientific knowledge and data through collaborative networks [63]. In this section, the uses and features of blockchain technology for biomedical research will be discussed.

The nature of biomedical research data implies that much of the data will be considered protected health information and/or personally identifiable information. All blockchain systems offer immutable data and an easily accessible audit trail. Most public blockchain implementations offer anonymity, but not data privacy, as all transactions are transparent and visible to the general public. With private or permissioned blockchains, a user must be given credentials to access the blockchain. Additionally, there are hybrid blockchains that can act as both a public and private chain at different times. In the vast majority of biomedical data use cases using protected health information or personally identifiable information, a private or hybrid system is necessary.

Data is retrieved from many sources in biomedical research, both from within a lab and between labs. In order to ensure reproducibility of the research, it is very important to have access to the data, be able to prove that the data is authentic, show a full history of what has happened to each data point (data provenance), and be able to show that the data is the same at the time of analysis as it was when it was collected (data integrity and reproducibility). Blockchain technology is ideal for this purpose as it can mathematically prove the integrity of the data and record the full history of a data point through hash chains. Provenance, in particular, is extremely important for research as it is directly related to data quality [64]. A blockchain-based system can record not only references to the original data but also every transformation and function applied to the data, leading to better reproducibility and potential detection of falsified data.

With the onset of the GDPR [65] in the European Union and changes to the Common Rule in the United States [66], consent has moved to the forefront as a concern for clinical trials data and other research data. Consent can be recorded in a mathematically provable and immutable fashion by blockchains. Smart contracts, which are self-executing contracts between two parties, have been utilized to enable quick and efficient recording of consent (as mentioned above by BG) [16, 67]. The use of smart contracts has also been outlined by the Pharmaceutical Users Software Exchange for health data mining in a way that patients/subjects can monetarily benefit from the use of their personal data for research purposes [68].

An increasing volume of biomedical research data is being directly obtained from devices. Distributed ledger technology has been shown to ensure the authenticity and integrity of data from wearables and IoMT devices [10] as well as in the more general Internet of Things (IoT) community [69]. With this increasing amount of data, it has also been shown that this technology can be used as a foundational layer for artificial intelligence and machine learning approaches to classify and analyze ‘big data’ [5].

Blockchain technology features exhibit significant promise for biomedical research applications, but many challenges remain. The technology is still evolving and enabling a wider range of use cases by the day. Amidst all this interest, it is important to remember that blockchain technology is not a panacea for all issues related to biomedical research data or open science. However, when combined with other technologies, it has the potential to provide a solution that can address the long-standing challenges related to reproducibility, integrity, and trust in biomedical research.

## Blockchain technology set to modernize the pharmaceutical supply chain?

### Tim K. Mackey (Fig. 1) and Maria Palombini (Fig. 8)

**Fig. 8:**
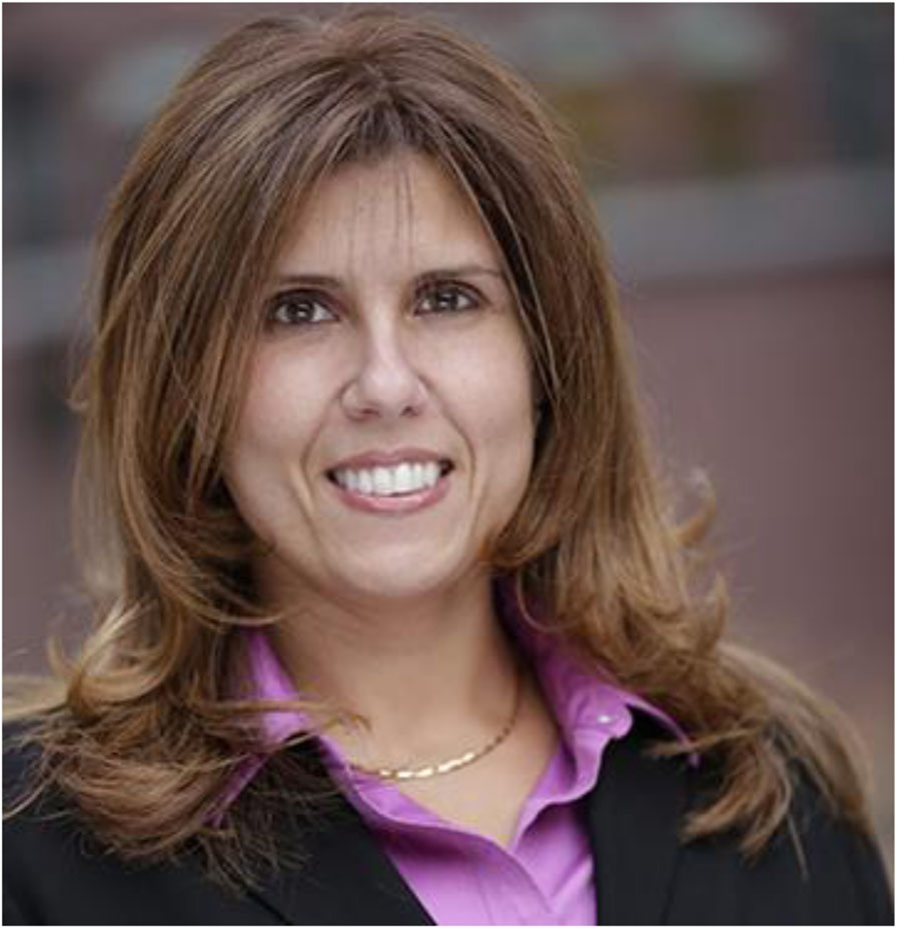
In 2017, Maria Palombini joined the IEEE Standards Association with a directive to build communities and advance initiatives to develop standards for enterprise adoption of emerging technologies. Her area of focus includes research on challenges and opportunities in integrating cutting-edge technologies such as artificial intelligence, Internet of Things, machine learning, virtual/augmented reality, smart materials and blockchain/distributed ledger technology (DLT) into the areas of pharmaceuticals, precision medicine, agriculture, and digital citizenship. She founded the Pharma Blockchain Initiative, which seeks to educate all stakeholders – patients, regulatory personnel, clinicians, pharmaceutical professionals, scientists, technologists – on the benefits and challenges of applying blockchain/DLT platforms to enable a patient-centric identity system that would benefit both the patient and critical operational areas of the pharmaceutical value chain. The work includes educating and building a global collaborative community that can reach consensus on technical standards and new protocol guidance required to enable industry buy-in for these emerging technologies and their breakthrough applications. Maria currently holds an MBA from the Rutgers Graduate School of Business and a BA and BS from Rutgers College at Rutgers University, the State University of New Jersey. She is an accomplished leader, public speaker, and writer, having presented at various industry events and published blogs and articles in trade publications

In the era of globalization, most supply chains now span multiple countries, actors, and products. Pharmaceutical supply chains are no exception and are characterized by complexity and fragmentation [70], including the different actors who participate in the drug supply chain such as manufacturers, wholesalers, repackagers, logistic providers, regulators, hospitals, pharmacies/dispensers and, of course, the end user patient [71]. They are global in nature, often involving trading partners in several different countries, oftentimes subject to a multitude of different trade, legal, and regulatory regimes. Throughout the health supply chain, the potential for drugs to experience breakdowns in management, quality, safety, or authenticity are a serious concern for public health [70, 72].

Blockchain has the potential to address many pharmaceutical supply chain challenges, though a prominent use case has been its application to combating falsified and substandard (i.e., counterfeit) medicines [12, 21, 70, 73]. Tackling this issue focuses on developing blockchain solutions that can enable trust and verification of supply chain data as medicines traverse the global supply chain, while also enabling participants to identify potential infiltrations of fake medicines through greater visibility of rogue supply chain transaction data [12, 21]. These systems could also act as a data architecture to enhance interoperability, track-and-trace, and e-pedigree requirements, all important characteristics of a modern digital supply chain [70].

Though different approaches have been explored, including by the Center for Supply Chain Studies, virtually all of these conceptual models focus on private or consortium business blockchains that adopt GS1 (open global standard for tracking healthcare products commonly used in barcodes) pedigree standards, with different degrees of permissions to transaction data on and off chain [74]. However, a major challenge to these drug safety- and regulatory-centric blockchains is governance, namely who will participate in the blockchain, how will data be validated, and most importantly, how will sensitive and confidential supply chain data be shared or not among participants? Many of these challenges necessitate building a consortium of interested parties that can agree upon these rules before a solution is even developed.

Several large companies and blockchain startups are active in the development of pharmaceutical supply chain blockchains [71]. Research and experimentation is also occurring, with one example of a published protocol that proposes a pharmacosurveillance blockchain system on a simulated network using distributed applications, smart contracts, and prototype instances built on both the Ethereum and Hyperledger fabric blockchain platforms [12]. However, design elements to ensure these blockchains are ‘fit-for-purpose’, scalable, and can withstand real-world testing remain in the early stages.

Uniquely, global pharmaceutical supply chains are also undergoing a period of policy modernization. Specifically, the FDA’s Drug Supply Chain Security Act outlines the necessary steps to implement an electronic, interoperable system to identify and trace prescription drugs distributed in the US [75]. This will enhance the FDA’s ability to protect consumers, including from drugs that may be counterfeit, stolen, contaminated, or otherwise harmful. The Drug Supply Chain Security Act also has a counterpart in the European Union, where the EU Falsified Medicines Directive requires measures to prevent falsified medicinal products from entering the legal supply chain by demanding safety features (including a unique product identifier and anti-tampering devices on packaging) to ensure identification and authentication [76].

Importantly, both of these policy instruments have regulatory requirements that can be facilitated via blockchain technology. For example, both policies require a unique identifier (through the use of serialization) to verify drug authenticity to deter counterfeit, or any other suspect medicine, from reaching patients. However, there are differences, with the EU Falsified Medicines Directive using a centralized approach where drug manufacturers upload serial numbers to a centralized EU regulator database, allowing distributors to connect and verify the authenticity of the drug. In the US, there is no centralized regulator database planned and it will be logistically challenging to have distributors integrate their data with that of pharmaceutical manufacturers. Thus, blockchain offers a potential solution to meet the needs of both markets to better ensure the integrity of serialization data and the provenance of pedigree and track-and-trace information. Blockchain systems could enable pharmaceutical manufacturers to share their serial numbers on the blockchain – decentralized and distributed with timestamps – where wholesalers, dispensaries, and prescribers would access to verify the provenance of the drug.

However, blockchain technology is not the panacea for combating the global criminal trade in falsified and substandard medicines. Though a noble goal, the unique challenges associated with the criminal nature of this trade, the presence of the grey market (i.e., access to medicines outside of the controlled supply chain such as the Internet), and the need for other forms of technology to appropriately authenticate and verify the physical product (e.g., analytical chemistry and anti-counterfeiting technology), may render blockchains ineffective when tested in the real-world and against nefarious actors [70, 77]. Thus, other use cases in the medicines supply chain vertical, including enhancing pharmaceutical public procurement, pharmacovigilance, recall management, returnable sales, enabling cold chain management integration with IoT, and even streamlining licensure and credentialing of supply chain actors, may have greater short- and even long-term utility than the fake medicines use case.

Adoption of blockchain into health supply chains is moving slower than in other industries (e.g., food supply and diamond supply chains). Illustrating some challenges, a study conducted by the IEEE Standards Association in 2017 on the state of blockchain adoption for the pharmaceutical supply chain found that three of the most frequently cited barriers of adoption among 300 qualified respondents (34% manufacturers, 33% distributors, and 33% retailers) were user buy-in/acceptance, integration into existing networks, and challenges (i.e., training) of implementing new technologies [78]. The cost of adoption and implementation is a primary challenge for supply chain stakeholders, as these blockchain solutions may not inherently drive revenue, but would instead enhance compliance and, hopefully, in the process lower costs or mitigate risk. These benefits may be hard to quantify, particularly in the context of fake medicines where the scope and prevalence of this activity is not well known [79]. This may position other cheaper technologies as more attractive alternatives to blockchain solutions.

Finally, the true value of a pharmaceutical blockchain may not be found in specific use cases. Instead, unlocking the potential of blockchain might best be used for broader goals of accelerating health supply chain modernization, unlocking data to improve supply chain performance and management, increasing transparency to enhance governance and accountability, moving towards regulatory harmonization of supply chain networks, and addressing issues regarding ‘last mile’ barriers in medicines access, quality, and affordability.

## Entering the genomics age with the help of blockchain technology

### George Church (Fig. 9), Dennis Grishin (Fig. 10), and Kamal Obbad (Fig. 11)

**Fig. 9:**
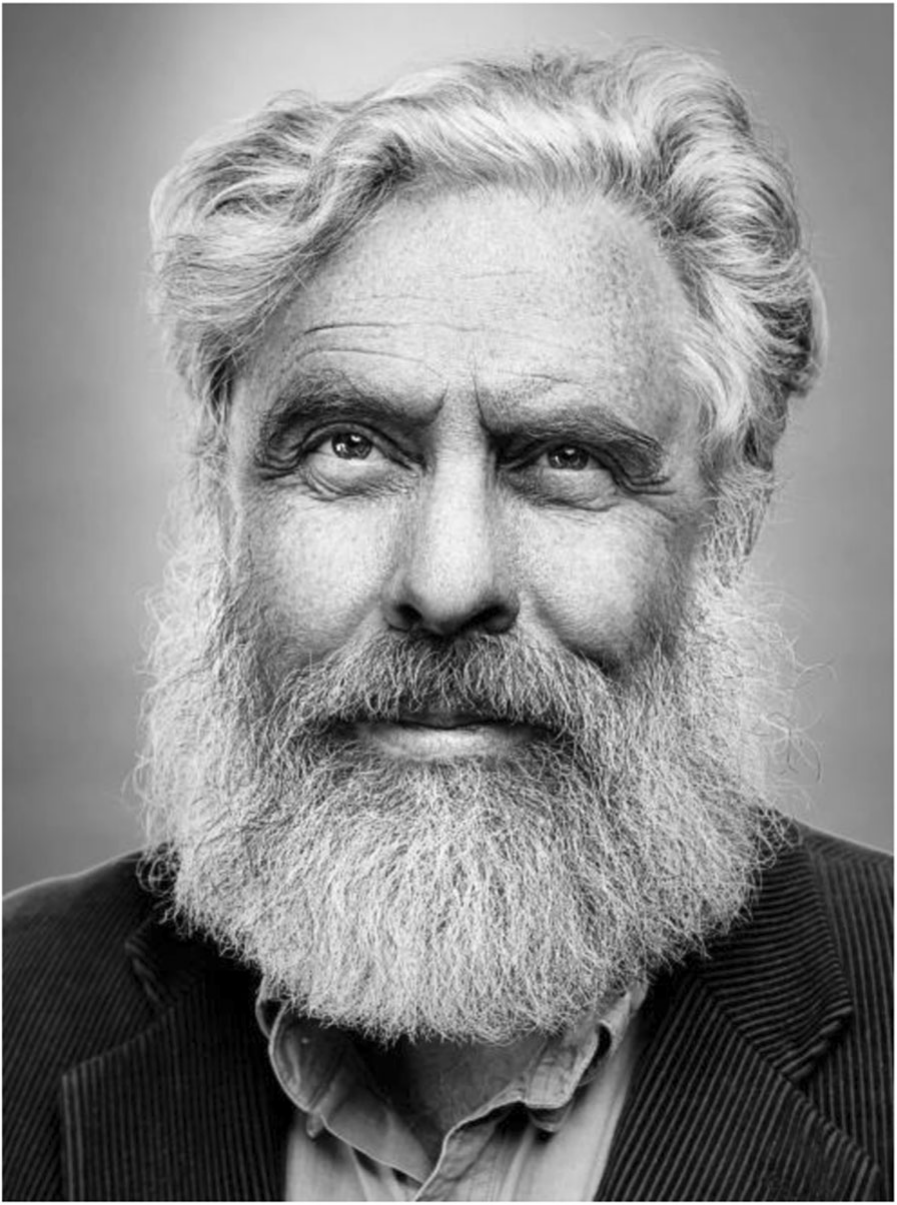
George Church is a Professor at Harvard and MIT. His lab developed methods used for the first genome sequence (1994) and million-fold cost reductions since (via NGS and nanopores), plus barcoding, DNA assembly from chips, and genome editing, writing and recoding

**Fig. 10:**
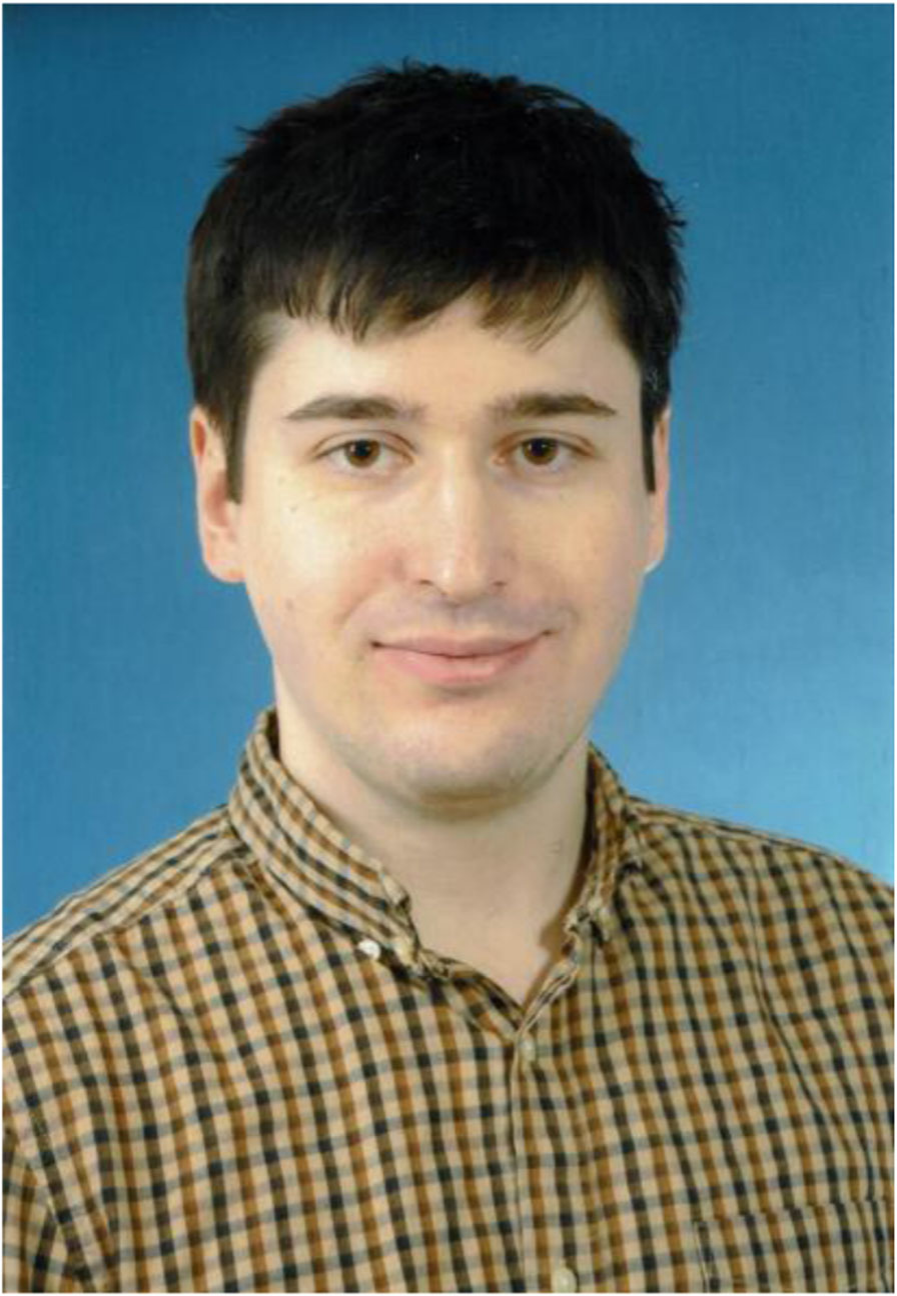
Dennis Grishin is CSO and co-founder of Nebula Genomics. He studied biology at the University of Freiburg and computer science at Harvard University and is a recipient of the German National Academic Foundation Fellowship. He is currently a Boehringer Ingelheim PhD Fellow in Genetics and Genomics at Harvard University

**Fig. 11:**
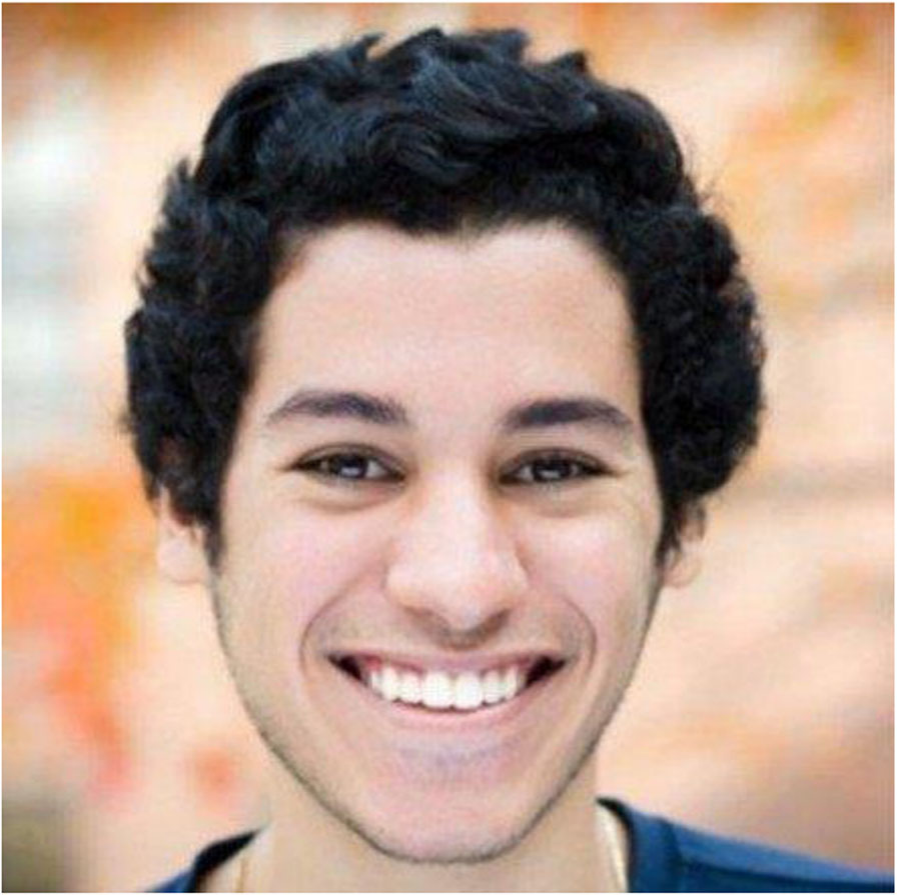
Kamal Obbad is CEO and co-founder of Nebula Genomics. He attended Harvard University, where he conducted research on neurological and embryological development. After Harvard, Kamal joined Google and worked within the research and machine intelligence organization. He is a recipient of the Gates-Cambridge Fellowship for the pursuance of graduate studies in advanced computer science

The development of next-generation DNA sequencing technologies has resulted in exponentially declining costs of human genome sequencing and has made personal genome sequencing affordable to many people. This unprecedented advancement has seemingly brought us closer to an age of genomic data-driven medicine and drug development. However, though there has been an increase in direct-to-consumer genetic testing, this transformation continues to be delayed due to lagging consumer adoption of whole genome sequencing and siloed datasets [80].

We have explored various approaches to popularizing personal genome sequencing and making genomic data more accessible. In 2005, we initiated the Personal Genome Project – a public genomic database – and have thus far recruited thousands of volunteers who have agreed to be sequenced and share their genomic data [81, 82]. In 2015, we helped make personal genome sequencing available for less than $1000, a price point that marked an important barrier broken towards an era of precision medicine. Our experiences have taught us two important lessons. Firstly, that few people are comfortable publicizing their personal genetic information and many are concerned about privacy policies of personal genomics companies [83]. Therefore, while the Personal Genome Project has made admirable progress, it has not experienced exponential growth. Secondly, most people do not value personal genome sequencing since the probability that they will immediately and significantly benefit from it is relatively low [84]. Thus, decreasing sequencing prices has not been enough to incentivize a mass adoption.

These lessons have led us to the conclusion that strong data privacy guarantees and novel incentive mechanisms are needed to drive genomic data generation and encourage data sharing. Blockchain technology can help catalyze a genomics data revolution by reducing personal genome sequencing costs, democratizing genomic data ownership, and enabling transparent genomic data sharing. This is made possible by two core properties of blockchain networks.

First, blockchain facilitates the creation of decentralized networks that enable participants to exchange data. The blockchain typically serves as an access control layer and can be used to implement a cryptocurrency that creates economic incentives to share data that is stored off chain. These general design principles can be applied to create a platform that enables individuals to control access to their personal genomic data and incentivizes data sharing with researchers and others who might benefit from access to the data. However, blockchain technology must be adapted to accommodate the unique challenges of genomic data. In particular, genomic data differs from many other data types in its scarcity, which is caused by the low adoption of personal genome sequencing. Thus, in addition to data access sharing, a genomic data exchange platform must also drive genomic data generation. Therefore, network protocols must be adapted to incorporate genomic data generators (DNA sequencing providers) as a third type of network participant. Together, genomic data generators, sellers, and buyers can create an economy that shifts sequencing costs from data sellers to data buyers and thereby drives genomic data generation.

Second, blockchain can act as a public ledger that immutably stores transaction records. This property can be leveraged to implement transparent consent management that incentivizes data sharing. To this end, data owners can add time-stamped entries to the blockchain that allows data buyers with permission to access their data. However, this functionality must also be ‘fit-for-purpose’ to ensure protection of highly sensitive personal genomic data. In particular, an effective consent management system for genomic data must require data buyers to reveal their identity (e.g., name and institutional affiliation) while data owners must be able to remain anonymous to protect their privacy. This can be implemented with a permissioned blockchain that allows only verified data buyers to access the network. Thus, transaction validator nodes must be operated by a consortium of collectively trusted ‘data guardians’. Such nodes can, for example, be operated by independent third-party, non-profit organizations whose aim is to support biomedical research and that can act as a fiduciary for a data owner’s interest.

Blockchain has been described as a revolutionary technology that will transform many different industries. Yet, for the nascent field of genomics, precision medicine, and pharmacogenomics, blockchain can be a truly enabling technology with an unparalleled impact.

## The future of the health blockchain: promising use cases and the importance of technical standards setting

### Maria Palombini (Fig. 8)

There is no doubt that for every touchpoint in the healthcare ecosystem there could be a blockchain application. The reason is simple – the healthcare ecosystem is nourished with data generation and sharing, from biomedical research in a lab with cell/tissue analysis all the way through to insurance payments when care is provided. However, in order to have a functional healthcare ecosystem, the data needs to be shared to all the critical parts so that patient care has continuity [85]. The necessity to share data throughout the ecosystem is what makes blockchain a viable application for healthcare.

The many features of blockchain technology lend themselves to one undisputable reality, namely the ability to evenly negotiate the tension between data sharing and privacy. For decades, healthcare delivery organizations, pharmaceutical companies, physicians, and health service providers have relied on policy to maintain a valuable asset, such as patient data, siloed and protected. In the absence of technology platforms that could guarantee an equal balance of patient data sharing and privacy, these organizations not only benefitted from leveraging the data but also compromised it.

The reality of the need to collaboratively share and maintain the privacy of healthcare data has resulted in 2 years of blockchain experimentation, with mixed results. This period of early health blockchain exploration has been characterized by the rise and fall of proof-of-concepts and pilot projects that have yet to enter into robust production and usability. However, there is now growing ‘consensus’ and progress on use cases (e.g., drug supply chain, clinical trials and research, and patient centric identity) deemed viable for blockchain and health, some of which have been covered in this Forum article.

In addition to these use cases, one of the greatest opportunities that blockchain offers is to accelerate precision medicine (as previously discussed by GC, DG, and KO). Specifically, patient-centric identity empowers the patient with rights to consent to, and choose how to, use their data in exchange for health services or even compensation. It also provides auditability of whom, when, and where personal health data are utilized. For example, based on our discussions with patient advocacy groups, patients who suffer from rare, chronic or terminal diseases, are more incentivized to find or contribute to a therapy or treatment. However, these same patients often do not have access to, or portability of, their healthcare profiles, which can inform clinical trial matching, access to potential experimental treatment, or aid in drug discovery.

Blockchain technology can address the issue of healthcare data silos that are provider-centric and not patient-centric by enabling open health data exchange markets driven by patients. These open health data markets will not be the average data warehouse; they will be populated with what is termed ‘V^3^ Data’ – validated, verified, and valuable. Market data will not be passively collected from mobile or internet searches or generic wearable data; rather, it would consist of clinically verified diagnostics, treatment outcomes, real-world evidence, genetics, DNA profiling, and more. Some examples of current and operational personal health data exchanges include Embleema, which launched the first patient-driven, HIPAA-compliant, health records blockchain aiming to solve the challenges associated with the collection and safe sharing of real-world evidence [86], and Shivom, which is creating a global platform for the secure storage and sharing of genomic data [87]. Thus, the open health data market creates many opportunities that were once considered impossible, including (1) placing the patient as the driver of data through empowerment and the right to consent to sharing their data, receiving compensation, and viewing audits of transactions; (2) creating a more competitive marketplace for smaller pharmaceutical and biotech companies that currently cannot afford to compete in clinical research and development because the cost of generating data out of trials is prohibitively expensive; and (3) providing access to much needed data to address challenges in population health and precision medicine.

However, in order to drive compliant and efficient blockchain adoption at larger scale in life science and health applications, technical data standards and regulatory policies need to be developed to ensure proper protocols and policies are created as it relates to distribution, management, and control of patient data [15]. Regulatory agencies do not create technical standards and standards organizations do not create policy; however, regulatory agencies are more likely to adopt policy and guidance on technologies that have market-driven, consensus-built technical standards. Therein lies the critical link between policy and standards setting.

Some have argued that technical standards can constrain innovation in uncertain markets [88]. The reality is that, if new technologies enter the market without some credibility, they will not be adopted for industrial nor consumer applications. Thus, market-driven and consensus-built technical standards directly address some of the uncertainties associated with the adoption of new technologies such as blockchain, including supporting interoperability with existing technology systems, reducing the cost of integration by removing the need for customization, establishing credibility (through consensus-built standards), enabling industry-wide adoption, driving competition through the creation of open-platform standards (‘platform agnostic’), and offering the ability to harmonize policy specifically around the use of technologies (where possible).

There are blockchain standards that have been published and/or are currently in development from both an industrial application and technical perspective [78]. Some of the currently published standards include the Chain Open Standard for Finance [89] and the Enterprise Ethereum Alliance [90]. Technical standards not only alleviate the barriers of wide-scale adoption of technologies such as blockchain in industrial applications but also enable the convergence of other cutting-edge technologies such as IoT, 5G, and artificial intelligence to interoperate with the blockchain. Specifically, blockchain standards for pharmaceuticals or any other health application cannot be written in absence of existing industry standards for other interfacing technologies or processes. For example, to develop a pharmaceutical supply chain track-and-trace blockchain standard, it would need to include GS1 Standards that provide a common language to identify, capture, and share supply chain data [91]. Additionally, blockchain for clinical trial data sharing will need to utilize the HL7 (Health Level Seven International) standards, which provide a framework (and related standards) for the exchange, integration, sharing, and retrieval of electronic health information [92]. Along with the release of HL7 Version 2, the FHIRChain (Fast Health Interoperability Records + Blockchain) was launched [93], which is a blockchain-based architecture for shared clinical data that can enable blockchain solutions focused on healthcare record management.

As expected, technologists and industry executives are focused on the now – successfully implementing a fully operational blockchain for an identified use case. However, standards organizations need to focus on developing solutions for the challenges that will arrive tomorrow. The roll out of multiple blockchains will require more standards as it relates to chain-to-chain interoperability, providing insight to regulatory agencies for establishing policies and guidance, and continuous education for industry stakeholders, as well as patients.

The greatest benefits of blockchain are yet to be realized. However, the outcomes of successful and failed blockchain pilots will eventually lead to the promise of patient-driven healthcare systems in the form of open health data markets and precision medicine, finally reaching the patient.
